# Industrial Gear Oils: Tribological Performance and Subsurface Changes

**DOI:** 10.1007/s11249-018-1013-2

**Published:** 2018-04-10

**Authors:** Aduragbemi Adebogun, Robert Hudson, Angela Breakspear, Chris Warrens, Ali Gholinia, Allan Matthews, Philip Withers

**Affiliations:** 1 International Centre for Advanced Materials (ICAM, Manchester Hub), The University of Manchester, Manchester, M13 9PL UK; 2 School of Materials, The University of Manchester, Manchester, M13 9PL UK; 3 BP Europa SE - Castrol Industrial Monchengladbach, Monchengladbach, Germany; 4 BP Technology Centre, Whitchurch Hill, Pangbourne, RG8 7QR UK

**Keywords:** Boundary lubrication, Gear oils, Surface chemistry, Subsurface microstructure, Mechanical properties

## Abstract

This study examined the tribological performance of three gear oils (Oils A, B and C), in relation to surface and microstructural changes. Oil A contains molybdenum dithiophosphate friction modifier, Oil B contains amine molybdate combined with zinc dialkyl dithiophosphate antiwear additive, while Oil C contains phosphonate and a commercial gear oil package. Following sliding tests of a hardened AISI 52100 steel ball on a spheroidized AISI 52100 steel disc, the worn surfaces were chemically studied using Raman and energy-dispersive X-ray spectroscopy. The tribological performance for each oil was different, likewise the nature of the tribofilm formed. After a 5 min sliding test, the hardness-depth profile of the worn surfaces was measured; also the cross-sectional microstructure was examined using scanning electron microscopy combined with focused ion beam preparation and transmission electron backscattered diffraction (t-EBSD) techniques. With Oil A, there was a relatively small increase in surface hardness (33% greater than that of the unworn surface), whereas with Oils B and C, the average hardness near the surface was 100% greater than that of the unworn surface. The cross-sectional microstructure using Oil A also differed from Oils B and C, which were quite similar. The result shows that with Oil A refinement of the ferrite grains spreads deeper into the material (> 10 µm), whilst with Oils B and C it was largely limited to 2–3 µm below the surface. It is concluded that the lubricant formulations and their associated tribofilms influenced the extent of deformation in the subsurface layers and consequently influenced the wear performance.

## Introduction

Formulated oils used in mechanical systems such as gears and bearings are primarily designed to sustain low friction and wear of moving parts. Minimizing friction and wear improves energy efficiency and extends the lifetime of the systems. To achieve this, friction and wear additives are included in the formulation of industrial lubricants. This class of additives is particularly important in boundary lubrication conditions whereby surfaces come in direct contact as a result of a high load and low speed combination. In this case, the lubricant is too thin to prevent surface asperities from touching. Without these additives functioning properly in the formulated oils, gears and bearings can fail in catastrophic ways.

During sliding of surfaces, these additives are surface active and contribute to the formation of a protective layer or tribofilm that prevents direct metal contact and lowers friction between the surfaces on the bases of its low shear strength relative to that of the metal [1]. Friction and wear additives can be subdivided into subcategories. These include friction modifiers, antiwear and extreme pressure additives. Other classes of additives usually added to formulate oils include corrosion inhibitors, antifoam, dispersants and detergents. The focus of this study will be on the friction and wear additives. The formation mechanism of these tribofilms and their properties determines the level of friction and wear protection that they provide initially and over time.

Friction modifiers such as organomolybdenum compounds (OMCs) react with metal surfaces to form molybdenum disulphide (MoS_2_) and other compounds such as sulphides and oxides [2–5]. Transmission electron microscopy (TEM) characterisation of tribofilms formed on surfaces worn in the presence of OMCs has shown that they contain nanosheets of MoS_2_ [6, 7] which adhere well to the surfaces. Low friction observed with these friction modifiers is attributed to the ease of sliding between single layers of MoS_2_ in the tribofilms formed [6]. MoS_2_ is Raman active, and the first-order Raman modes are $E _{2g}^{2}$, *E*_1*g*_, $E_{2g }^{1}$, *A*_1*g*_ observed around 34, 287, 383 and 409 cm^−1^, respectively [8]. The $E _{2g}^{2}$ and *A*_1*g*_ modes have peaks with the highest intensity and can easily be used to identify the presence of MoS_2_. Commonly used antiwear and extreme pressure additives in industrial applications include sulphur–phosphorus compounds, molybdenum–sulphur compounds and ZDDP (zinc dialkyldithiophosphate). ZDDP additive is the most widely used antiwear additive. A comprehensive review of the history and mechanism of ZDDP has been reported by Spikes [9]. ZDDP can decompose on rubbing surfaces to form a rough pad-like tribofilm of glassy zinc phosphate/polyphosphate [10, 11] material with thickness of 50–200 nm. This tribofilm is effective at reducing wear by acting as a sacrificial layer with a faster rate of formation than the rate of removal. During sliding of surfaces under high load, surface asperities cannot be completely separated. Plastic deformation and/or fracture of the asperities is inevitable. Tribofilms are able to reduce friction and can influence plastic deformation of the surface asperities depending on their properties. Lower friction at the surface will reduce the level of frictional strain induced in the subsurface and play a significant role in wear reduction [12]. Frictional strain during sliding is usually localised very near the surface and continues to accumulate with more cycles of sliding. Under significantly high load and sufficient number of sliding cycles, most ferrous metals form a refined grain structure a few nanometres in depth [13]. This layer can eventually delaminate to form plate-like wear debris [14].

Friction and wear performance of different industrial oils have been widely studied and reported in the literature. An established approach to understanding the mechanisms by which industrial oils perform in boundary lubricated sliding is examining the properties of the tribofilm formed and surface topography. There is a limited amount of studies that have investigated the relationship between tribological performance, the nature of tribofilm formed and the subsurface changes. One of those who have studied this relationship is Reichelt et al. [15]. They studied the surface film formed during triboaction and the subsurface layer (or tribomutation layer) that formed beneath the surface film. Their work showed that the wear protection provided by formulated oils is a function of the combined properties of the induced surface film and the underlying tribomutation layer. Cao et al. [16] reported in a recent study that tribofilms had an effect on wear by influencing the plastic flow of the nanograined structures generated near surface of the contacts. Two different oils produced very different tribofilms. Although they produced similar levels of friction coefficient in a 2 h sliding test, there was a large disparity in the level of wear protection. The difference in the level of wear protection was attributed to the ability of the tribofilm to hinder or promote grain rotation. A softer film would allow for more degrees of rotation of the nanocrystalline layer, as a result reducing wear significantly. The concept of surface films influencing the deformation behaviour of surfaces dates back to the 1990’s. One of the earliest research was conducted by a Russian scientist Peter Rehbinder [17]. He and his colleagues in 1928 observed that the presence of a lubricant can significantly affect changes that take place in a solid contact. The presence of oleic acid on the surface increased the ability of a mineral crystal to deform in a plastic manner. Similarly, in 1969, Donald Buckley [18] showed that the mechanical behaviour of calcium fluoride crystal was sensitive to extremely small concentrations of surfactant. Although this concept has been investigated by some [15, 16, 19] in recent times, there is still more room for exploration particularly in the development of industrial lubricants.

The aim of this study was to investigate the relationship between tribological performances of three industrial gear oils (via their tribofilm) and subsurface mechanical and microstructural changes.

## Materials and Methods

### Lubricant

The test lubricants were three ISO VG 320 gear oils, which all have the same kinematic viscosity of 320 ± 32 mm^2^/s at 40 °C. Oil A is used in wind power gearboxes, Oil B in heavy industrial applications (such as mining vehicles) and Oil C is a multifunctional gear oil used in a variety of applications. The complete formulation of the lubricants includes the base oil, and the complete additive mix are shown in Table 1. Oil A is formulated with synthetic polyalphaolefin (PAO) base oil and an additive package that includes molybdenum dithiophosphate (MoDTP) friction modifier, mixed sulphonates and an antifoam. Oil C is also formulated with synthetic PAO base oil with a different additive mix, containing phosphonate, alkylated naphthalene, antifoam and a commercial gear oil package. Oil B is formulated with a mineral base oil and additive package that combines a friction modifier (amine molybdate), an antiwear additive (ZDDP), extreme pressure sulphurised additive and metal sulphonate (corrosion inhibitor). These three oils with different base oil-additive mixes were selected with the intention of generating different types of tribofilms with different tribological properties.

**Table 1:** Industrial formulated oils and their base oil/additive mix

	Oil A	Oil B	Oil C
Base oil	Polyalphaolefin (> 90%)	Group 1 base stock mix (> 85%)	Polyalphaolefin (> 80%) Alkylated naphthalene (5-15%)
Additives	Molybdenum dithiophosphate (MoDTP)—(< 5%)	Amine molybdate complex (< 5%) ZDDP (< 5%) Sulphurised—extreme pressure additive	Phosphonate (< 1%) commercial gear oil package (< 5%)
	Mixed sulphonates—corrosion inhibitor (< 0.5%) Copper—corrosion inhibitor (< 0.5%)	Sulphonate mix—corrosion inhibitor (< 5%)	
	Antifoam—trace	Antioxidant (< 1%) Methacrylate polymer—pour point depressant (< 5%)	Antifoam—trace

### Tribotesting

Sliding tests were carried out using a high-frequency reciprocating rig (HFRR, PCS Instruments, London, England). The test configuration is a ball-on-disc, where the disc is held stationary, submerged in the test lubricant. Load is applied on the test ball which oscillates linearly on the disc. For the entire sliding test, a load of 9.8 N is applied on the test ball and it oscillates on the disc with a frequency of 50 Hz for a stroke length of 1 mm providing average Hertzian contact pressure of 0.96 GPa. All tests were carried out at 80 °C by heating up the lubricant bath and maintaining the temperature throughout the test. The temperature of 80 °C was selected to match the temperature at which gear oils typical operate in industrial systems. The test duration was varied between 5 min and 2 h. Each test was carried out three times to give average values of friction coefficient. The HFRR system measures and stores the friction coefficient continuously throughout the test. Additionally, electrical contact resistance (ECR) is monitored. ECR gives an indication of whether or not a separating film is formed between the ball and disc. A 15-mV potential is applied between the ball and disc which form a resistance referred to as the contact resistance; this potential is also applied to a balance resistor which is in series with the contact resistance and forms a potential divider [20]. A series resistance of 1000 Ω is selected in the HFRR control software. The change in potential between the contacts (referred to as electrical contact voltage) is a measure of the ‘contact resistance’ in comparison with the balance resistor. A zero electrical contact voltage indicates a direct metal–metal contact, and no electrical contact resistance between the contact and the formation of a fully insulating film means that the electrical contact voltage reaches a maximum voltage of 15 mV.

### Ball and Disc

The test ball and disc were made of AISI 52100 steel. The ball is 6.0 mm in diameter with hardness of 11.7 ± 1.0 GPa and surface finish or roughness (Ra) of less than 0.05 µm. The test disc is 10 mm in diameter and 3 mm thick, with a hardness of 3.3 ± 0.2 GPa and surface roughness of about 0.02 µm. Before each test, the test ball and disc were cleaned with toluene and acetone. After each experiment, the test samples were cleaned with heptane to remove the residual oil. The test discs were further degreased with soapy water and ethanol prior to characterisation in the scanning electron microscope.

### Wear Measurement

A KEYENCE VK—X200 3D confocal microscope was used to scan the profile of the wear scar on the test disc, followed by post-processing of the measurement with KEYENCE VK Analyser. Tilt correction was applied to adjust for the slight tilt of the sample on the microscope stage. Wear volume was calculated by the product of the wear scar length and the cross-sectional areas. The average of 10 2D profiles along the wear scar was used to determine the cross-sectional area. The Ra of the worn surfaces was measured by applying a cut-off wavelength (*λ*_c_) of 0.025 µm to the primary profile which eliminate the influence of the longer wavelength components.

### Raman Spectroscopy

A Renishaw 1000 microscope system was used to analyse residual tribochemical products on the wear scars. The wavelength of the laser source was 514 nm. All scans were carried out with an Olympus objective lens which gives a laser spot size of about 1 µm. Raman spectra were obtained from the wear scar at 1 s exposure and 20 accumulations to reduce the signal-to-noise ratio.

### SEM–EDX

A scanning electron microscopy (SEM) FEI Quanta 650 FEG equipped with energy-dispersive X-ray (EDX) spectroscopy detector was used to characterise the wear surface topography and map the elements present on the surface. Surface imaging and EDX mapping were performed with accelerating voltages of 5 and 30 kV, respectively.

### Nanoindentation

The hardness of the near surface after sliding tests was measured using the MTS Nano Indenter XP. The continual stiffness measurement (CSM) mode makes it possible to continuously measure mechanical response with depth as an indenter tip penetrates the surface [21, 22]. The Diamond Berkovich indenter tip was displaced into the material at a constant strain rate of 0.05 s^−1^ to a depth of 2 µm. Hardness measurements were taken on the as-received surface and the three worn surfaces.

### SEM–FIB

**Fig. 1:**
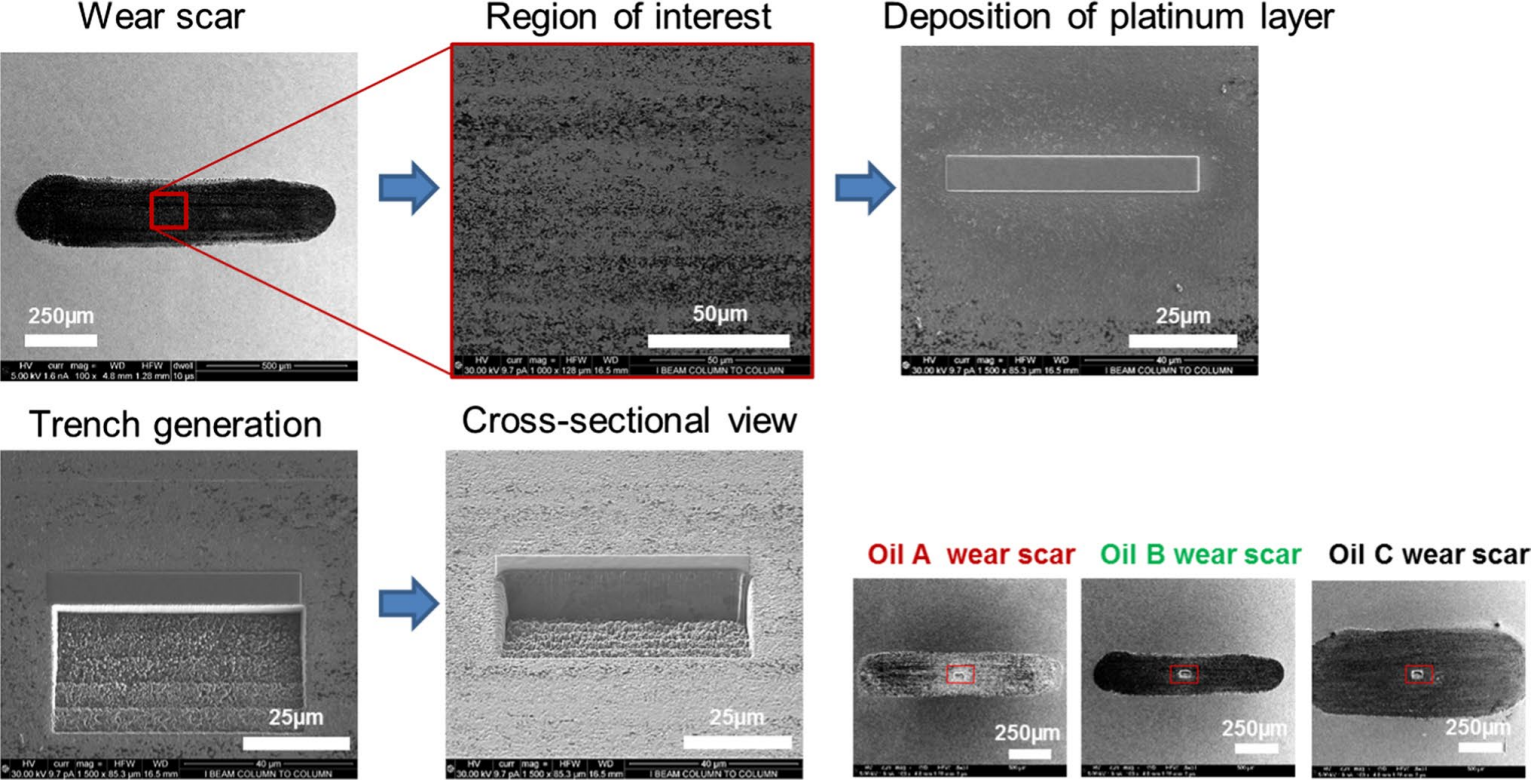
SEM micrographs showing the process of subsurface microstructural examination using the SEM–FIB technique

A SEM equipped with FIB was used to characterise the subsurface microstructure of the spheroidized AISI 52100 steel discs before and after sliding test with the three oils. The study was carried out using a FEI Nova NanoLab 600i system. The dual beam system has secondary electron and focused ion beams. Electron images were taken with voltage of 5 kV and current of 16 nA. Ion imaging was taken with voltage of 30 kV and beam current of 9.7 pA. Imaging of the subsurface microstructure was done using ion channelling contrast technique [23]. With this technique, grains appear either dark or bright based on their orientation. The region of interest is set as the centre of the scar for the three worn surfaces. This is one of the advantages of using the FIB technique, as it allows site-specific analysis. The FIB process (Fig. 1) involves firstly depositing a platinum (Pt) layer on the surface to protect the region of interest; a trench is generated by ion beam milling to reveal the cross-sectional microstructure.

### Transmission-EBSD

T-EBSD in comparison with the conventional EBSD technique provides improved spatial resolution as a result of a reduced interaction volume of the electron beam in the sample [24]. This is particularly useful in resolving nanosized grains. An FEI Magellan 400 XHR SEM equipped with NordlyNano EBSD detector was used to take EBSD measurements. Using FIB lift-out technique [25] in an FEI Nova NanoLab 600i dual beam system, a thin FIB lamella (< 200 nm) was generated from the cross sections (Fig. 9b, c) and mounted in transmission geometry before characterisation with the SEM. An accelerating voltage of 30 kV was used with probe current of 1.6 nA and a step size of 20 nm. The Kikuchi patterns were indexed using Aztec 2.2 software. EBSD data processing was done using Channel 5 software suite developed by Oxford Instruments HKL.

## Results and Discussion

### Friction and Wear Performance

In this section, friction and wear performance are discussed in relation to tribofilm formation on the surface. Although all the additives in the mix play a role in the performance of the formulated oils, this discussion focuses on the role of the friction and wear additives. Both Oils A and B contain organmolybdenum friction modifiers, MoDTP and amine molybdate, respectively. Oil B contains ZDDP antiwear additive in addition to amine molybdate friction modifier. Oil C contains sulphur-and-phosphorus-based antiwear and extreme pressure additives.

**Fig. 2:**
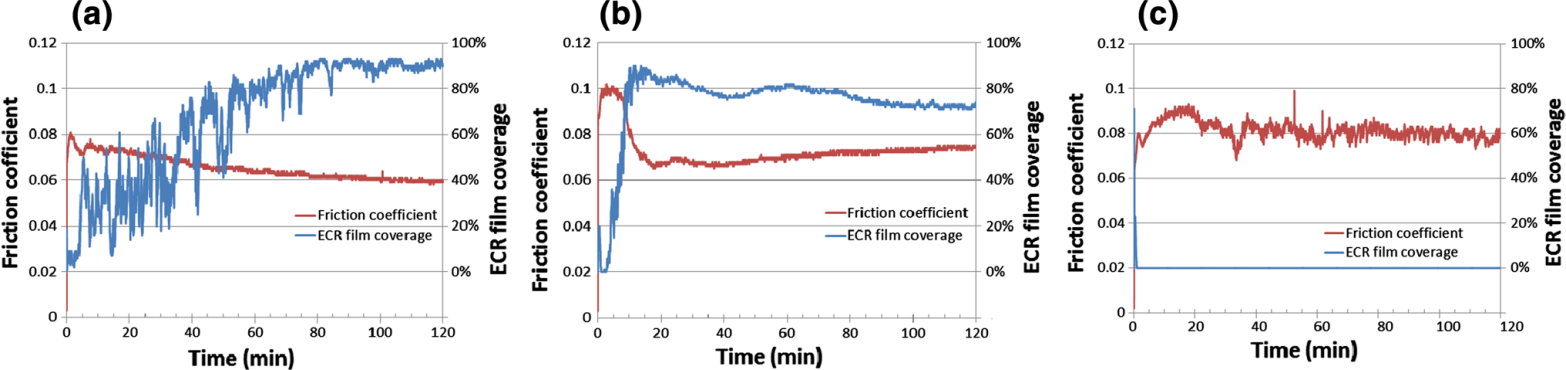
Plots of friction coefficient and ECR film coverage for **a** Oil A, **b** Oil B and **c** Oil C

**Fig. 3:**
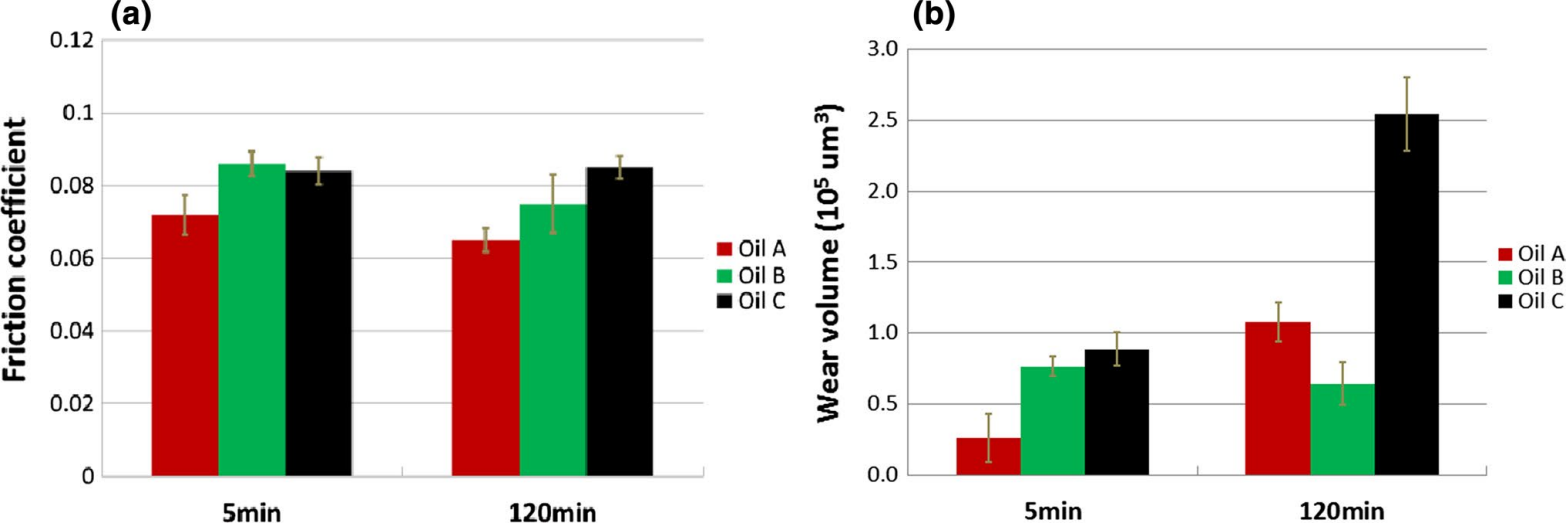
Average **a** friction coefficient and **b** wear volume for the three oils after 5 and 120 min of HFRR sliding test

**Fig. 4:**
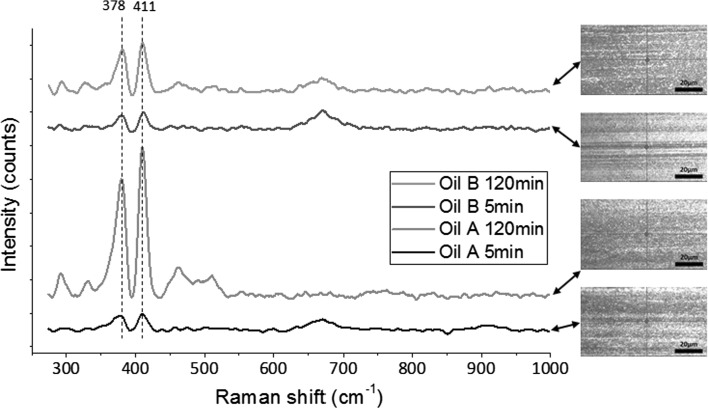
Raman spectra obtained from surfaces lubricated by Oils A and B after 5 and 120 min (2 h). The red dot at the centre of the crosshair in the optical images shows the location each spectrum was taken from

**Fig. 5:**
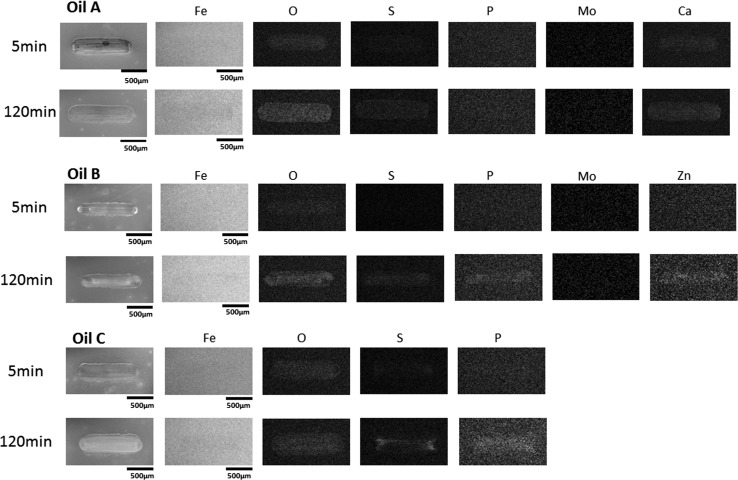
SEM images and EDX maps of wear scars lubricated by Oils A, B and C after 5 min and 2 h

The three fully formulated oils were tested in the HFRR tribometer for durations of 5 min and 2 h. Three tests were run for 5 min, and another three were run for 2 h continuously. The recorded mean value of friction coefficient at the end of the 5 min tests was averaged and is reported in Fig. 3a. The same goes for the 2 h tests. After the test, wear measurements were taken from the wear scars generated on the test discs since there was no evidence of wear on the test balls. At the start of the 2 h sliding test, Oil A provides a low friction coefficient of about 0.08 and this drops slightly to 0.07 within 5 min (Fig. 2a). Raman spectroscopy results indicated that MoS_2_ was present on the worn surface at this time (Fig. 4) as evident by $E _{2g}^{2}$ and $A_{1g}$ peaks at 378 and 411 cm^−1^, respectively. The EDX elemental map shows the surface contains oxygen, sulphur and calcium after 5 min (Fig. 5). One of the additives included the formulation of Oil A is calcium sulphonate. The elemental print of calcium on the wear scar indicates that the calcium sulphonate is surface active and contributes to the tribofilm formed. The formation of MoS_2_ between sliding metal surfaces is known to be the cause of significant reduction in friction [2, 4, 5, 26]. From 5 min to the end of the 2 h test, the friction coefficient continues to drop steadily to reach 0.06. MoS_2_ remains present on the worn surface after 2 h of sliding as evidenced by the Raman spectra in Fig. 4. The elemental map of the wear surface generated after 2 h of sliding (Fig. 5) shows an increase in the intensity of oxygen, sulphur and calcium. As the friction coefficient drops, the ECR plot (Fig. 2a) shows the build-up of an unsteady insulating film. At the end of the 2 h test, wear has increased substantially with Oil A (Fig. 3b). This suggests that the insulating film was either insufficient to protect against wear or contributed to the increase.

When testing Oil B, the friction coefficient was 0.1 for the first 10 min of the sliding test and this was then followed by a sharp drop to 0.065 (Fig. 2b). This drop in friction coefficient has been attributed to the formation of a low friction film [27, 28]. The ECR result for Oil B shows a sharp rise in electrical contact resistance at the start of sliding suggesting a film forms quickly at the contact. Raman spectroscopy was used to scan the surface for evidence of MoS_2_ formation on the surface after 5 min and 2 h. The results presented in Fig. 4 indicate that MoS_2_ was formed after 5 min and remains present at the end of the 2 h test. The EDX elemental map of the surface shows that oxygen is present on the surface after 5 min; and after 2 h of sliding, sulphur, phosphorus and zinc are present in addition to oxygen. The presence of zinc and phosphorus after 2 h of sliding coupled with the relatively low wear obtained with Oil B suggests the formation of zinc phosphate film which is known to play a significant role in the low wear performance when ZDDP is present [10, 11].

Similar to Oil A, the friction coefficient of Test Oil C is initially at about 0.08, but the friction coefficient profile for Oil C is rougher than that of Oils A and B, as evident by the short order spikes and dips. This suggests that the tribofilm, if present, is very unstable, and this is also implied by the lack of electrical contact signal throughout the 2 h test (Fig. 2c). The EDX elemental map (Fig. 5) shows that oxygen and a smaller proportion of sulphur are present on the surface after 5 min. After 2 h, the surface contains oxygen, sulphur and phosphorus.

The average friction coefficient and wear volume for each of the oils at 5 and 120 min are presented in Fig. 3. Oil A provides the lowest friction after both 5 and 120 min of sliding. This can probably be attributed to the formation of low shear MoS_2_ tribofilm at the contact, which prevents direct metal to metal contact and reduces friction. However, in the 2 h test, wear increased considerably. The result shows that Oil A provides relatively good friction performance, but average antiwear performance, particularly over longer periods. Oils B and C provide higher average friction coefficients of 0.087 and 0.089, respectively, after 5 min of sliding test compared to Oil A. After 2 h of sliding test, Oil B provides the lowest wear and forms an insulating film that covers about 75% of the contact surfaces (Fig. 2). Oil B appears to form a complex tribofilm consisting of MoS_2_ and a phosphate constituent. This complex tribofilm provides relative low friction and excellent wear performance. The elemental map of the surface worn by Oil C for 2 h suggests that a S–P-based film might have formed on the surface although this may not have reflected on the ECR plot due to its conductive nature. The roughness of the friction coefficient plot and the substantial increase in wear after 2 h suggests that any film formed was probably too unstable to adequately protect the surface, as evident by the relatively high wear.

**Fig. 6:**
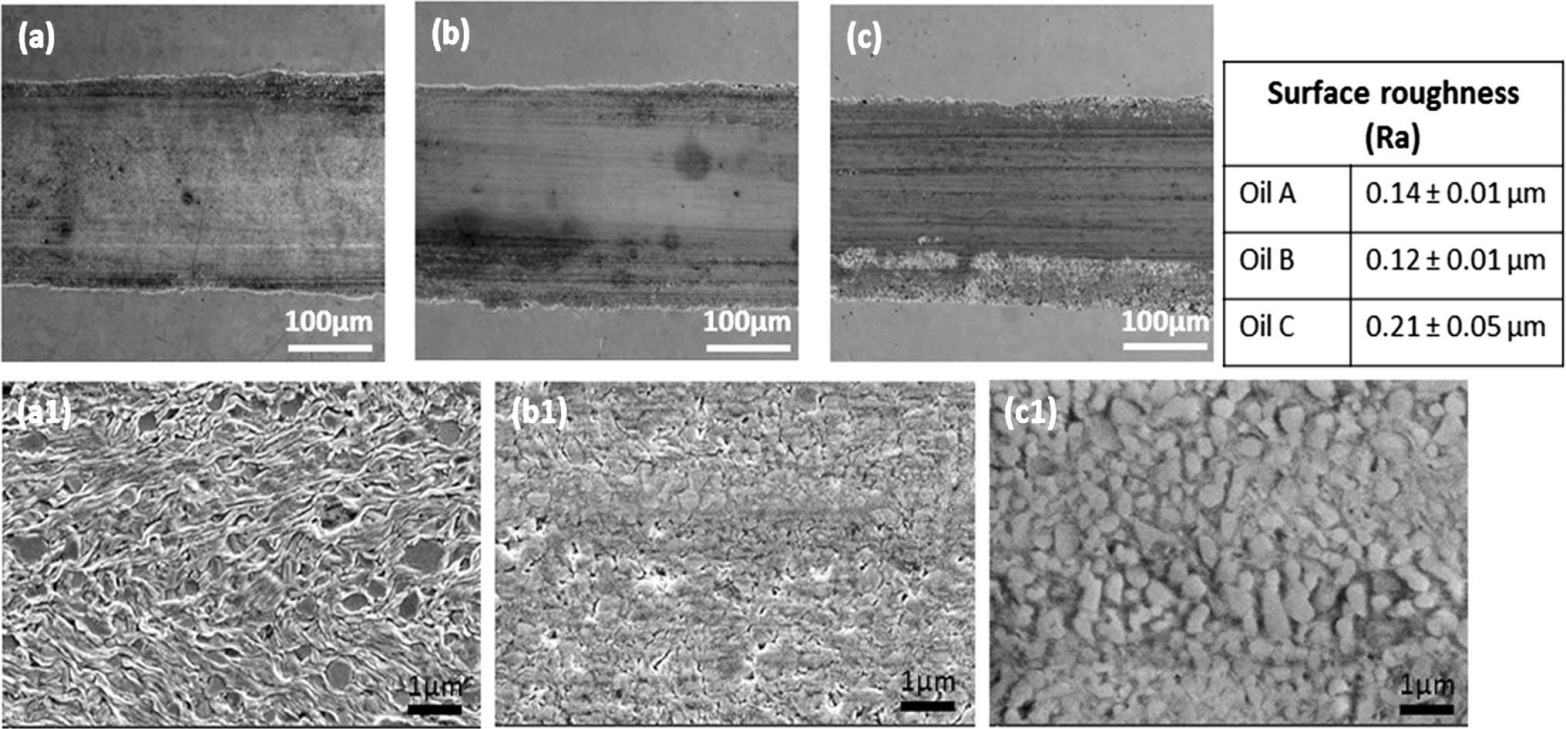
SEM images of wear surfaces generated from 2 h sliding test with Oil A (**a**, **a1**), Oil B (**b**, **b1**) and Oil C (**c**, **c1**)

**Fig. 7:**
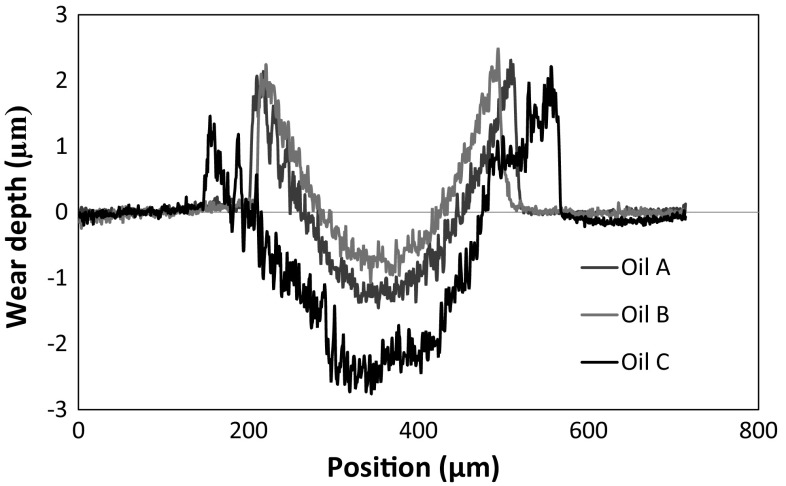
Cross section profiles of the wear scars generated from 2 h sliding tests with Oils A, B and C

The surface topography of the worn surfaces after 2 h is presented in Fig. 6. The worn surfaces from the three oils (Fig. 6a–c) show evidence of plastic deformation caused by ploughing of a hardened AISI 52100 steel balls on the softer AISI 52100 steel discs. Figure 7 shows the cross section profile of the wear scars generated with the three oils which corresponds to the wear result (Fig. 3b) after 2 h sliding. For Oil A, the higher magnification micrograph (Fig. 6a1) shows the softer ferrite matrix organised in a wavy pattern around the hard cementite particles, which has a darker contrast. The surface worn by Oil B (Fig. 6b1) appears smooth and homogenous, which was confirmed by the surface roughness measurement. For Oil B, the Ra is 0.12 µm, which makes it the smoothest of the three surfaces. On the other hand, the surface lubricated by Oil C appears to be rough with a Ra value of 0.21 µm; it is the roughest of the three surfaces. Also, the surface appears to be densely populated with cementite particles. The cementite particles appear to be sticking-out, which could be the reason for the high surface roughness value measured. This combined with the high density of cementite particles possibly suggests that most of the soft ferrite matrix surrounding the hard cementite particles has been worn away.

### Subsurface Changes (Mechanical and Microstructural)

Section 3.1 focuses on how the tribological performance of the gear oils relates to the nature and extent of tribofilm formed and also the surface topography. However, the tribological performance of the oils is also a function of changes taking place beneath the surface. The aim of this section is to investigate the effect of friction and wear performance on subsurface changes (mechanical and microstructural) and any correlation that might exist. Subsurface characterisation was conducted on the worn surfaces generated after 5 min of sliding in the HFRR tribometer. Since the test ball is approximately 3 times harder than the test disc (substrate), plastic deformation and wear are expected to occur entirely on the substrate. No wear was observed on the test ball. Therefore, investigation of subsurface changes was conducted on the spheroidized AISI 52100 steel substrate.

Frictional energy generated during sliding of surfaces is dissipated in many ways. Most of the energy is dissipated as frictional heat and will raise the temperature of the surfaces significantly even in well lubricated surfaces [29]. The rest of the energy is partitioned into other processes including: formation and shearing of tribofilm, transformation of the microstructure (plastic deformation) and the process of wear [30]. How frictional energy is dissipated during sliding has been the subject of previous studies [30–32]. These studies highlight the importance of considering just how frictional energy is dissipated to different process taking place in a tribosystem. Evaluating the energy balance in the tribosystem during the sliding test is outside the scope of this study. Consequently, analysis of the results in this study has been simplified by assuming that the percentage of frictional energy dissipated to tribofilm formation and shearing, plastic deformation and wear is equal for the three oils. Oil A provided the lowest average friction coefficient of 0.073 during the 5 min sliding test, while Oils B and C provided similar levels of friction at 0.087 and 0.089, respectively. Based on the assumption made, the higher friction coefficient of Oils B and C would equate to higher stored energy in the subsurface layer and wear.

**Fig. 8:**
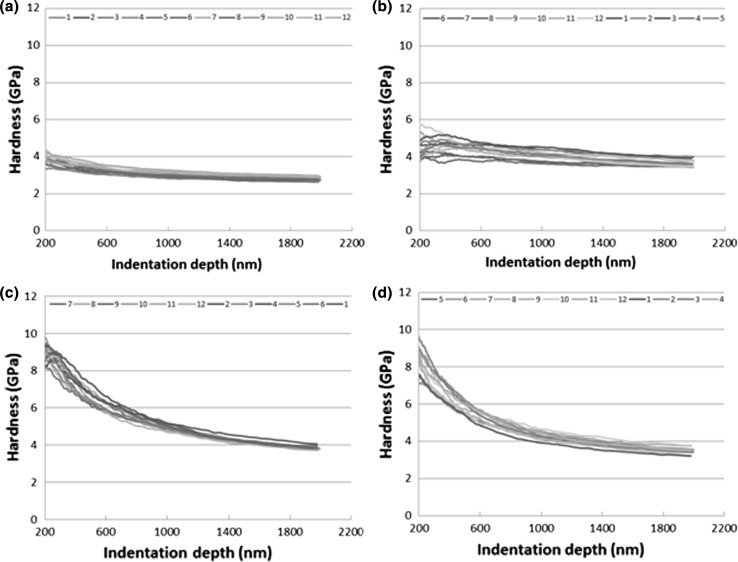
Variation of hardness with depth on **a** the unworn surface, and the worn surface after sliding test of 5 min with **b** Oil A, **c** Oil B and **d** Oil C

Nanoindentation technique was used to measure the hardness-depth profile for the unworn substrate and those worn using Oils A, B and C to a maximum depth of 2 µm. The hardness-depth profile for the unworn surface in Fig. 8a shows that the average hardness is approximately 3 GPa and was relatively homogenous up to the measured depth of 2 µm. The hardness-depth profiles for Oils B and C in Fig. 8c and d, respectively, are similar and different to that of Oil A. With Oils B and C, there is significant rise in hardness towards the top surface from 3.5–4 GPa at 2 µm below the surface to 7.5–10 GPa at 200 nm. However, with Oil A there is a relatively low increase in the hardness from 3GPa in the unworn substrate to average of 4 GPa. Another distinction with Oil A is that the increase in hardness is uniform with depth up to 2 µm. The significant hardening of the subsurface layer with Oils B and C in comparison with Oil A is perhaps the first indication that there might be a correlation between the higher level of friction coefficient of Oils B and C to the higher level of energy stored in the subsurface layer.

**Fig. 9:**
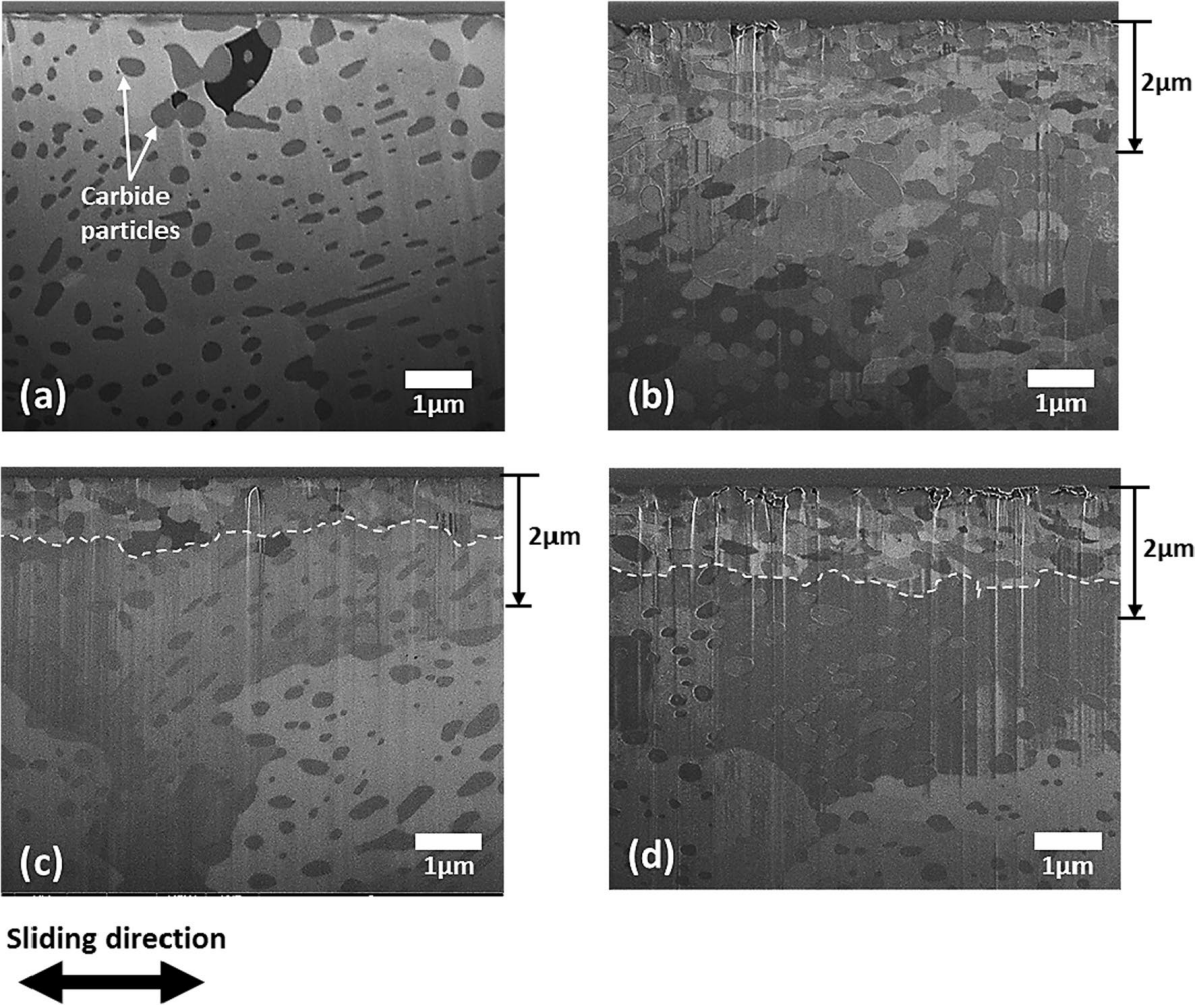
Microstructures beneath **a** the unworn surface, and the worn surface after sliding test of 5 min with **b** Oil A, **c** Oil B and **d** Oil C

Further examination of subsurface transformation was carried out using FIB technique combined with ion channelling contrast imaging (ICCI) to reveal subsurface microstructure. This was preferred to the conventional approach of sectioning, grinding and polishing, because of the relatively small size of the wear scar (approximately 1.5 mm by 0.5 mm). Additionally, this technique makes it possible to analyse roughly the same location (centre of the wear scar) for each of the three different samples (Fig. 1). Figure 9a shows the subsurface microstructure of the substrate in the unworn state which consists of cementite (FeC) particles uniformly distributed in large ferrite grains with average grain size of 15 µm. As surfaces plastically deforms during sliding, dislocations or defects are generated and accumulate in the ferrite grains. As sliding progresses, these dislocations with strain energy associated with them begin to form dislocation tangles (DT) or dislocation dense walls (DDW) [33]. Intersecting DDW subdivides the original ferrite grains into dislocation cells or refined blocks, and with the accumulation of more dislocations, these cells evolve into sub-grains with small mis-orientations between them. With progressive refinement, of the ferrite grains, the subsurface layer is substantially work-hardened and explains the increase in hardness in Fig. 9. The presence of the cementite particles in ferrite facilitates refinement of the large ferrite grains [34].

**Fig. 10:**
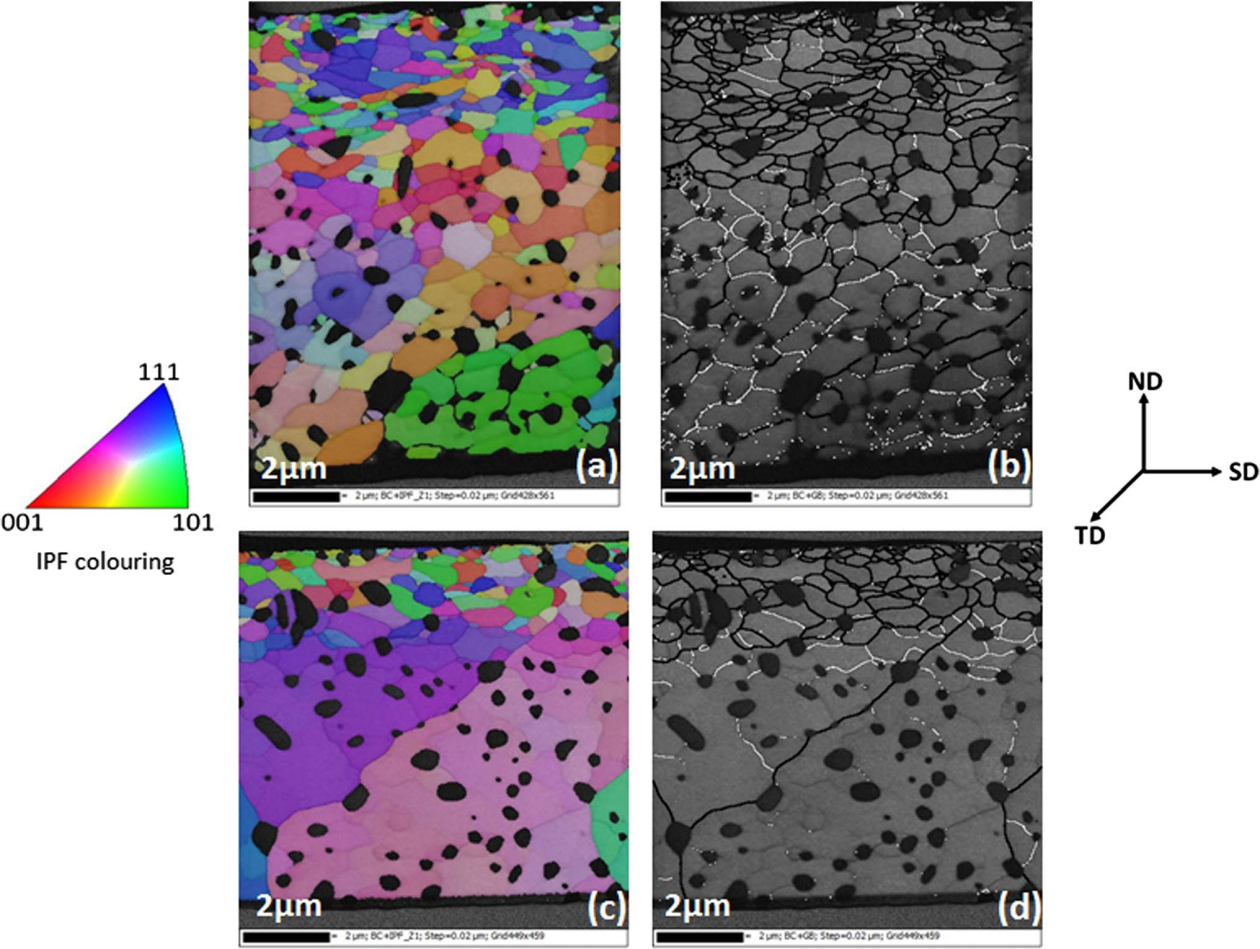
Cross-sectional EBSD images of FIB lamella extracted from the centre of the worn surfaces: **a**, **c** inverse pole figure map for Oils A and B, respectively, **b**, **d** grain boundary superimposed on band contrast map for Oils A and B, respectively. Black lines represent high-angle GB’s (> 10°), and white lines represent low-angle GB’s (2° ≤ *θ* ≤ 10°)

The cross-sectional microstructures of surfaces lubricated by Oils B and C (Fig. 9c, d) are similar and different to that of Oil A (Fig. 9b). With Oils B and C, the subsurface microstructure reveals a top layer (< 3 µm thick) of refined ferrite grains above large ferrite grains with the cementite particles uniformly distributed. However, the subsurface microstructure below the surface worn with Oil A (Fig. 9b) reveals refined ferrite grains of varying sizes surrounded by cementite particles to a large depth greater than 10 µm below the surface. To better characterise the grain structures observed in Fig. 9b and c, the transmission electron backscatter diffraction (t-EBSD) technique described in Sect. 2.9 was employed. The process involved extracting out a thin slice of about 100–150 nm from the cross section shown in Fig. 9b and c. The thin slice was then examined in transmission mode. This analysis was conducted for the cross sections of only Oils A and B since the cross-sectional microstructures of Oils B and C are quite similar. The EBSD inverse pole figure maps (Fig. 10a, c) allow clear observation of the grain size, grain orientation which is colour-coded and the grain size distribution with depth. One obvious distinction between the microstructures of Oils A and B in Fig. 10a and c, respectively, is the difference in the depth of the refined grains. Grain refinement is localised very near the surface with Oils B and C and corresponds to the hardness profiles in Fig. 8c and d, respectively. However, with Oil A grain refinement spreads deeper into the material. Also with Oil A, some of the grains within the top 3 µm below the surface appear elongated and below 3 µm the average size of the refined ferrite grain appears larger than those in the top layer for Oil B. The subsurface microstructures in Figs. 9 and 10 correspond to the hardness profiles in Fig. 8, and yet again, there is similarity between subsurface changes with Oils B and C. This is perhaps a second indication that there might be a correlation between higher friction coefficients obtained with Oils B and C to the localisation of strain near the surface, whereas with Oil A providing lower friction coefficient the strain accumulated to a larger depth below the surface. Friction coefficient influences the subsurface stresses during sliding. The zone of maximums stress moves from the subsurface closer to the surface as friction coefficient increases [35, 36]. This might explain why with Oils B and C with higher friction coefficient strain is localised nearer to the surface as evidenced by the hardness-depth profile and subsurface microstructures. Strain localisation can be induced by a stress gradient developing in the subsurface layer during mechanical processes [37].

Going by the earlier assumption made that the percentage of frictional energy partitioned into tribofilm formation and shearing, plastic deformation and wear process are equal during sliding with the three oils, higher wear (Fig. 3b) would be expected with Oils B and C which both have higher average friction coefficient (Fig. 3a) in comparison with Oil A after 5 min of sliding. Whilst that was the case in this study, it is not always so. It is clear from previous studies [16, 19] that although different oils can produce a similar steady-state friction coefficient during boundary lubricated sliding this does not always correlate to similar levels of wear. Nevertheless, subsurface microstructure generated during boundary lubrication sliding can be related to the process of wear. Nanocrystalline layers typically formed below the surfaces under boundary lubrication play a crucial role in generating wear particles [38] and the extent of wear [39, 40]. Buscher et al. [38] in their study of nanocrystalline layer and wear particle generation found that the size of wear debris during tribo-process corresponds to the nanocrystalline grain size. Therefore, they suggest that the wear particles are generated from nanocrystal torn off from the subsurface layer. Higher wear with Oils B and C might be linked to significant localisation of strain very near the surface forming fine ferrite grains near the surface in comparison with Oil A where the strain accumulation spreads deeper into the material. Perhaps as the refined grains gets smaller near the surface the boundary between the fine ferrite grains and the hard cementite particles weakens forming voids and eventual delamination of wear debris.

The only variable in the sliding tests conducted for this study was the lubricants and their corresponding additives mix. This will influence the nature and extent of tribofilm formed during sliding as discussed in Sect. 3.1. The tribofilms formed from the different oils during the sliding test influences how much friction is generated in the tribosystem, how friction evolves and is dissipated into other simultaneous processes such as microstructural transformation and wear. Although it was assumed that the frictional energy generated during sliding is partitioned into other simultaneous processes in the same manner for the different oils; by simply changing the lubricant used, the dynamics of how friction is dissipated into heat and other simultaneous process will likely change. There are several characteristics of the tribofilm formed that can influence the dynamics. Some of these characteristics have been investigated by other researchers in the past including: mechanical properties (shear strength, hardness, etc.) of the tribofilm [41–45], how quickly forms and stabilizes [46–48] and its durability [49–52].

## Conclusions

This experimental paper has investigated the importance of combining surface characterisation (surface chemistry and topography) with subsurface examination (mechanical and microstructural changes) to better understand the mechanisms by which industrial oils perform tribologically in boundary lubricated sliding. Hence, the following conclusions are drawn:

- The combined results of ECR film formation generated by the HFRR tribometer during the sliding test with ex situ chemical characterisation (Raman and EDX spectroscopy) of the worn surface improve understanding of the tribofilm formation process and its influence on tribological performance.
- The gear oil formulation determines the nature of tribofilm formed on the surfaces during sliding; consequently, it influences the type of transformation that takes place in the metal subsurface layer mechanically and microstructurally.
- After 5 min of sliding, Oil A provided lower friction and wear compared to Oils B and C. When compared to the original surface, Oil A produced a 33% increase in the surface hardness after sliding. Also, the depth of deformation evident by refined grains near the surface is > 10 µm. Conversely, Oils B and C produced over 100% increase in surface hardness and localised deformation to a depth of 2–3 µm below the surface.
- Tribosystems are complex due to the inter-relationship between simultaneously occurring processes such as generation of frictional heat, tribofilm formation and shearing, plastic deformation and wear. This makes it difficult to make direct correlations for example between frictional performance and subsurface changes.
- The nature of subsurface changes in tribological processes influences the extent of wear therefore to better understand how industrial lubricants and the tribofilms they form influence wear it is important to investigate their influence on subsurface changes.
